# An LC Wireless Passive Pressure Sensor Based on Single-Crystal MgO MEMS Processing Technique for High Temperature Applications

**DOI:** 10.3390/s21196602

**Published:** 2021-10-03

**Authors:** Pinggang Jia, Jia Liu, Jiang Qian, Qianyu Ren, Guowen An, Jijun Xiong

**Affiliations:** Science and Technology on Electronic Test and Measurement Laboratory, North University of China, Taiyuan 030051, China; pgjia@cqu.edu.cn (P.J.); jialiu@nuc.edu.cn (J.L.); b1806019@st.nuc.edu.cn (J.Q.); b1806005@st.nuc.edu.cn (Q.R.); anguowen@nuc.edu.cn (G.A.)

**Keywords:** LC wireless passive, high temperature, pressure sensor, single-crystal MgO, wet etching, direct bonding

## Abstract

An LC wireless passive pressure sensor based on a single-crystalline magnesium oxide (MgO) MEMS processing technique is proposed and experimentally demonstrated for applications in environmental conditions of 900 °C. Compared to other high-temperature resistant materials, MgO was selected as the sensor substrate material for the first time in the field of wireless passive sensing because of its ultra-high melting point (2800 °C) and excellent mechanical properties at elevated temperatures. The sensor mainly consists of inductance coils and an embedded sealed cavity. The cavity length decreases with the applied pressure, leading to a monotonic variation in the resonant frequency of the sensor, which can be retrieved wirelessly via a readout antenna. The capacitor cavity was fabricated using a MgO MEMS technique. This MEMS processing technique, including the wet chemical etching and direct bonding process, can improve the operating temperature of the sensor. The experimental results indicate that the proposed sensor can stably operate at an ambient environment of 22–900 °C and 0–700 kPa, and the pressure sensitivity of this sensor at room temperature is 14.52 kHz/kPa. In addition, the sensor with a simple fabrication process shows high potential for practical engineering applications in harsh environments.

## 1. Introduction

High-temperature pressure measurements play an important role in many applications, such as turbine engines, high-speed aircraft, nuclear power plants, and other aerospace applications [1,2,3,4]. To date, various types of sensing devices have been developed for monitoring pressure in high-temperature environments by using different sensing mechanisms and sensitive materials, including piezoelectric pressure sensors (LiNbO_3_, Ca_2_Al_2_SiO_7_, and YCOB) [5,6,7], piezoresistive pressure sensors (silicon-on-insulator, SiC, and poly-Si) [8,9,10], optical fiber pressure sensors (silica fiber and photonic crystal fiber) [11,12], and wireless passive pressure sensors (alumina and zirconia ceramics) [13,14,15]. 

Wireless passive inductive capacitive (LC) resonance sensors are good candidates in high-temperature or rotation environments because of their lower operating frequency, simple manufacturing, and near-field coupling distance [16]. So far, single and multi-parameter wireless passive sensors based on a low- and high-temperature co-fired ceramics (LTCC)/(HTCC) technique have been reported [17,18,19,20,21,22,23]. Tan et. al proposed an LC wireless passive sensor based on LTCC, an imidization process, and screen-printing technology for simultaneous multi-parameter sensing, and demonstrated that the sensor can work in an ambient environment of 25–200 °C, 24–90% RH, and 70–220 kPa, with a pressure sensitivity value of 3.25 kHz/kPa [24]. Lin et. al presented a wireless passive pressure and temperature-integrated LC resonant sensor, which adopted the low Young’s modulus materials as the sensor substrate. The pressure sensitivity is 1.16 kHz/kPa within the measurement range of 140–850 kPa at 500 °C [25]. However, compared to ceramics, single-crystal materials are more suitable for high-temperature sensors in long-term practical applications due to their stable physical and chemical properties. In our previous work, an LC-type pressure sensor based on sapphire was reported, but the pressure sensitivity was 10 kHz/kPa and the operating temperature was only 600 °C [26]. 

Single-crystalline magnesium oxide (MgO) is an attractive composition material for high-temperature sensors because of its superior mechanical, low dielectric constant, low high-frequency dielectric loss, ultra-high melting point (2800 °C), and thermal properties at elevated temperatures [27,28]. In this paper, we propose a single-crystal MgO MEMS-based LC wireless passive pressure sensor for harsh monitoring. The sensor consists of a variable capacitance, an inductance, and a vacuum-sealed cavity. First, a wet chemical etching method was used to fabricate a cylindrical cavity structure on the MgO surface. Then, direct bonding of the etched MgO and bare MgO was performed to obtain a sealed cavity as the sensor substrate. Finally, the wireless coupling test was performed on a high-temperature, high-pressure experiment platform to verify the sensor performance. In addition, the thermal stress mismatch within the sensor structure was reduced by developing the MEMS technology of MgO. This sensor can be batch-produced and is expected to be used in ultra-high-temperature environments. 

## 2. Sensor Design and Principle

**Figure 1:**
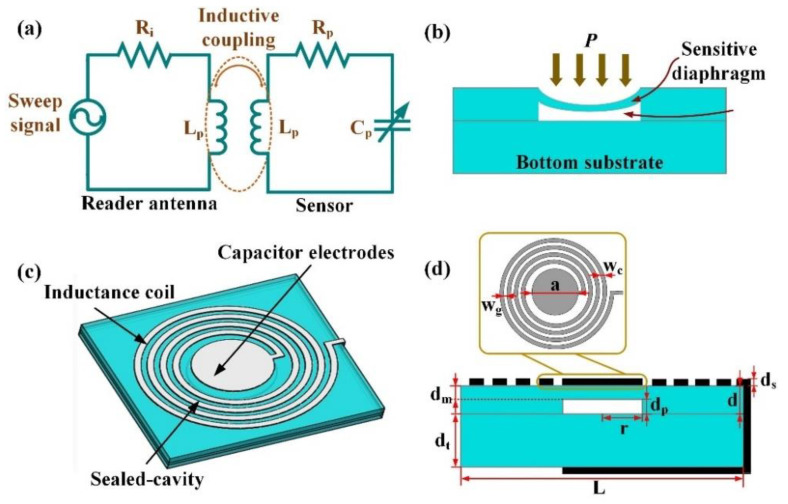
Schematic diagram of sensor operation. (**a**) Illustrative equivalent circuit schematic model of LC measurement. (**b**) Pressure sensing principle. (**c**) Structure of the sensor. (**d**) 3D model of the sensor.

The equivalent circuit schematic of the sensing system is illustrated in Figure 1a. The planar inductance coils and reader antenna of the resonant sensor are coupled when the reader antenna is close to the sensor. The sensor is resonant when the frequency of the sweep signal is the same as the self-resonance frequency of the sensor. Afterwards, the input impedance and phase of the reader antenna changes with the external environment. The sensor resonant frequency can be obtained by tracking and extracting the change in the resonance information of the reader antenna, and can be expressed as [29]

(1) $$f_{0}=\frac{1}{2\pi \sqrt{L_{p}C_{p}}}$$

where *L_p_* and *C_p_* are the constant inductance and variable capacitor of the sensor, respectively. 

As for the pressure loading, the formula for calculating the resonant capacitor *C_p_* can be expressed as [30]

(2) $$C_{p}\left(P\right)=\frac{C_{p}\left(0\right)}{\sqrt{\frac{\varepsilon_{r}d_{0}}{\varepsilon_{r}d_{p}}}}+\tan^{-1}\sqrt{\frac{\varepsilon_{r}d_{0}}{\varepsilon_{r}d_{p}}}$$

(3) $$C_{p}\left(0\right)=\frac{\varepsilon_{0}d}{d_{p}}+\frac{\varepsilon_{r}\varepsilon_{0}\left(L^{2}-4d^{2}\right)}{d_{p}}$$

where *d_p_* is the depth of the pressure cavity, $d_{m}\;$is the thickness of the pressure diaphragm, L is the side-length of the substrate, $\varepsilon_{r}$ and $\varepsilon_{0}$ are the relative permittivity of the substrate dielectric and vacuum permittivity, respectively, and $\varepsilon_{0}\;$= 9.65 × 10^−12^ F/m.

As shown in Figure 1b, when external pressure is applied to the diaphragm, the maximum deformation occurs in the center of the diaphragm. The center deflection $d_{0}$ of the sensitive diaphragm with the applied pressure can be expressed as [31]

(4) $$d_{0}=\frac{3\left(1-\mu^{2}\right)P}{16Ed_{m}{}^{3}}{\left(r\right)}^{4}$$

where r is the radius of the cavity, *P* is the pressure applied on the diaphragm, *E* is Young’s modulus, and μ is Poisson’s ratio. For MgO, *E* = 256.6 GPa and *μ* = 0.157. Furthermore, the maximum deformation of the diaphragm and the pressure measurement range are restrained by the structure parameters of the sensor. Therefore, the desired pressure sensitivity and measurement range can be obtained by adjusting the radius of the cavity and the thickness of the sensitive diaphragm.

Figure 1c shows the 3D model of the sensor, which consists of an embedded sealed cavity, inductance coils, and capacitance electrodes. The cross-section schematic diagram of the sensor is shown in Figure 1d; the overall structure of the sensor is a square with a side length of 20 mm and a height of 0.6 mm. The mechanical deformation and output response frequency of the sensor can be optimized through numerical simulation and electromagnetic simulation. Considering the performance, size, and coupling distance of the sensors, the sensor parameters are shown in Table 1.

## 3. Sensor Fabrication

**Figure 2:**
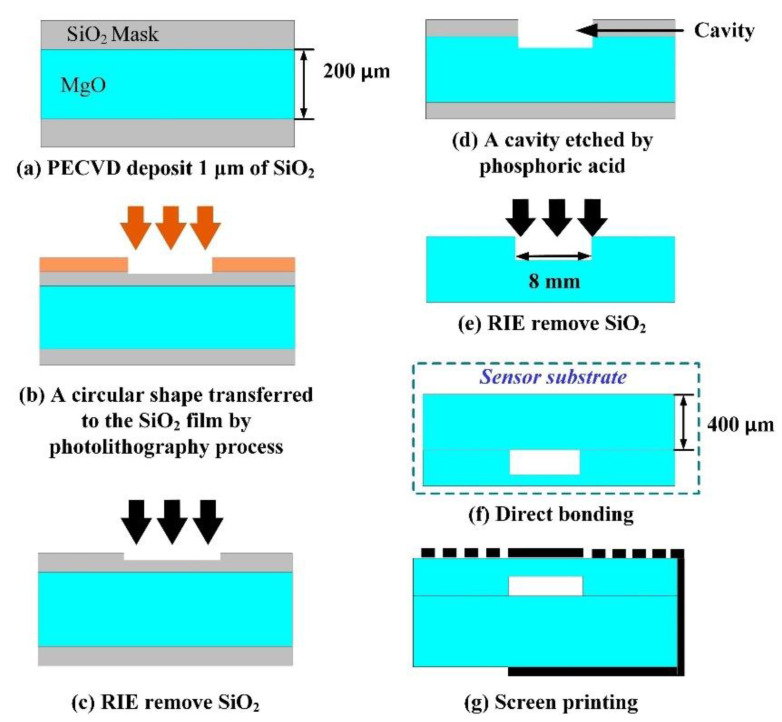
The fabrication process of the single-crystal MgO LC wireless passive pressure sensor.

The fabrication process of the LC wireless passive pressure sensor mainly consists of three steps: wet chemical etching, direct bonding, and screen-printing, as shown in Figure 2. MgO {100} with a side length of 20 mm and thicknesses of 400 μm and 200 μm were selected as the bottom substrate and sensitive diaphragm layer, respectively.

First, a cylindrical deep cavity was fabricated on MgO (thickness of 200 μm) by using the wet chemical etching method, as shown in Figure 2a–e. In our previous work, we analyzed the effect of different etchants on the surface morphology, surface roughness, and surface elements of MgO [32]. Phosphoric acid is the preferred etchant and the etching parameters are a concentration of 50% and a temperature of 120 °C. After a duration of 17 min, a cylindrical cavity with a diameter of 8 mm and a depth of approximately 74 μm (in theory) was obtained. Correspondingly, the thickness of the pressure-sensitive diaphragm was 126 μm. Then, direct bonding of the etched MgO and bare MgO (thickness of 400 μm) was performed, using a surface activation-assisted high-temperature annealing direct bonding method, as shown in Figure 2f. According to our previous research on the bonding strength of MgO/MgO under different annealing conditions [33], the parameters of direct bonding for the sensor substrate were determined to be a pressure of 4 MPa, a temperature of 1300 °C, and a duration of 140 min. Then, the vacuum-sealed cavity was produced.

**Figure 3:**
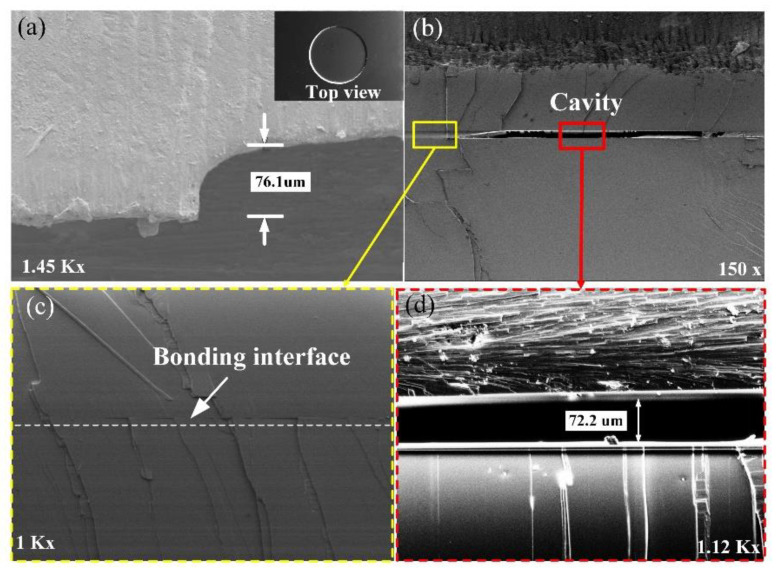
(**a**) The cross-selection SEM image of the cavity. (**b**–**d**) SEM images of the bonding interface and cavity at different magnifications.

To evaluate the etching and bonding quality, the cavity and bonding interface were observed by a scanning electron microscope (SEM, SU5000, HITACHI, Tokyo, Japan), as illustrated in Figure 3. The cross-sectional SEM image shown in Figure 3a indicates that the cavity length is 76.1 μm, which is consistent with the theoretical calculation. From Figure 3b–d, it can be seen that the cavity is complete, and the bonding interface is smooth and void-free, which contributes to the sealing of the pressure capacitor cavity. 

**Figure 4:**
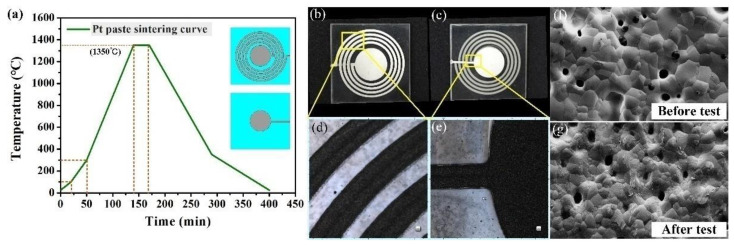
(**a**) Sintering curve of Pt conductor. (**b**,**c**) The top and bottom layers of the sensor. (**d**,**e**) The partially enlarged details of the inductors. (**f**,**g**) SEM images of Pt before and after high-temperature pressure testing.

Finally, a high melting point metal platinum electronic paste (Pt) (ESL5541-A, ESL Ltd., King of Prussia, PA, USA) was used to print inductance coils. Capacitor electrodes and inductor coils were printed on both sides of the sensor substrate with Pt paste by a screen printing technique. Specifically, the bonded MgO sample was aligned and pasted to the printing screen and the Pt was printed onto the MgO surface. The silk-screen had the geometrical parameters of a thickness of 25 μm and a resolution of approximately 320 meshes. After screen printing, the bonded structure was introduced into a muffle furnace to adhere the Pt layer to the sensor substrate. Figure 4a shows the sintering curve of the Pt conductor. The sensor fabrication was accomplished after the above processes, and the images of the fabricated sensor are shown in Figure 4b,c. The enlarged images of the inductance coils observed by optical microscope are shown in Figure 4d,e. It can be seen that the edge of the inductance coil is clear and complete. At the same time, the SEM results, shown in Figure 4f,g, demonstrate that there was no change in the microstructure of the Pt before and after the high-temperature pressure test.

## 4. Experiments and Results

**Figure 5:**
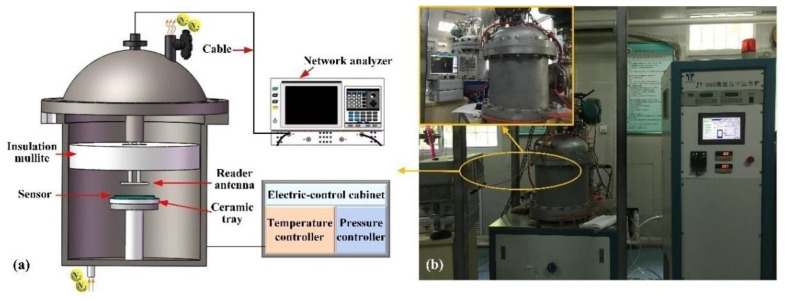
Temperature–pressure composite measurement platform. (**a**) Working principle of pressure measurement system. (**b**) High-temperature pressure experimental platform.

A high-temperature and pressure composite measurement platform (JT-300, Chengdu Jiangtai Co., Ltd., Chengdu, China) was used to verify the feasibility and performance of the wireless passive pressure sensor, as shown in Figure 5. The sensor was placed on the ceramic tray with a hole, which ensured that the temperature and pressure were accurately applied to the sensor. A copper reading antenna was placed at a vertical distance of 2 mm from the sensor, and the other end of the reader was connected to a network analyzer via a cable for gathering the resonance frequency, which is suitable for wireless sensing applications in small, confined spaces and high-temperature mechanical rotating structures. In addition, the cable can be immune to high-temperature damage by being placed above the insulation mullite. The temperature (accuracy of 0.5 °C) in the tank was controlled by the electric control cabinet and displayed in real time. Below the tank was a N_2_ entrance, and the pressure with the accuracy of 0.01 kPa applied to the sensor was controlled by adjusting the amount of N_2_.

**Figure 6:**
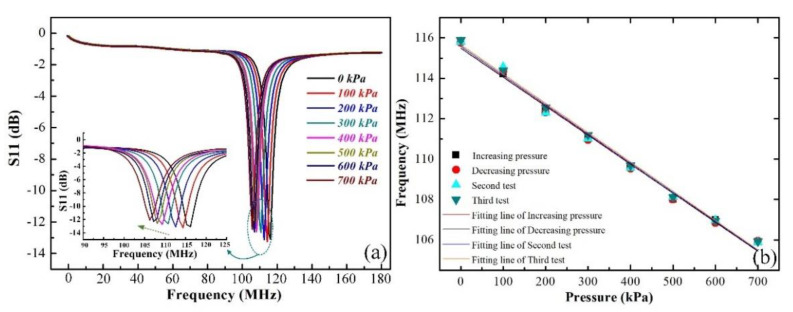
Pressure test results of the proposed sensor at room temperature. (**a**) The pressure versus resonant frequency curve under 0 kPa–700 kPa. (**b**) The frequency responses to pressure during the pressure increasing and decreasing process.

First, we increased the pressure from approximately 0 kPa to 700 kPa at intervals of 100 kPa at room temperature to verify the resonant frequency responses to pressure. The results shown in Figure 6a indicate that the frequency of the sensor decreased from 116.672 MHz to 106.509 MHz due to the sealed-cavity deformation during the pressure loading process, and the pressure sensor realized a high sensitivity of 14.52 kHz/kPa at room temperature. Furthermore, we also investigated the resonant frequency as a function of the pressure increasing and decreasing at room temperature to illustrate the hysteresis and repeatability of the sensor. As can be seen from Figure 6b, the coincidence of the frequency curves from the three experiments proves that the sensor has a repeatable response, and the resonant frequency is approximately linear with the pressure. Moreover, the hysteresis error is about 0.94% from the first increasing and decreasing pressure experiment.

**Figure 7:**
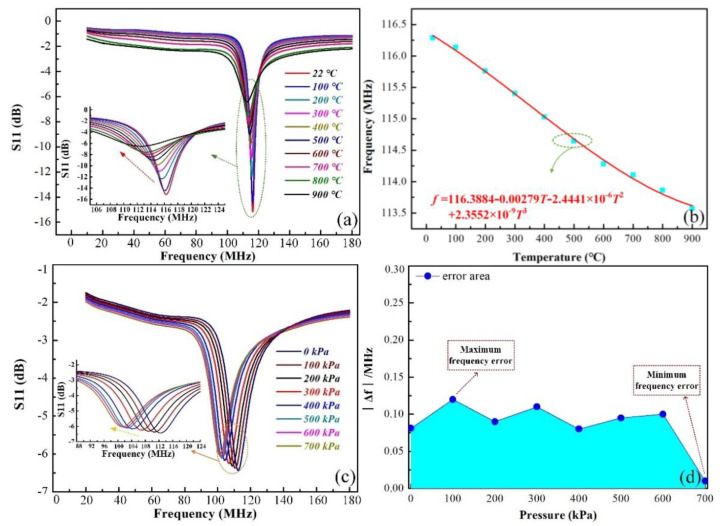
(**a**) The resonant frequency changes with the temperature under 0 kPa. (**b**) The zero drift of the sensor. (**c**) The pressure versus frequency from 0 kPa to 700 kPa at 900 °C. (**d**) The frequency error of the sensor at 900 °C.

Next, we increased the temperature from 22 °C to 900 °C at intervals of 100 °C under a pressure of approximately 0 kPa. The relationship between the amplitude of S11 and the temperature is shown in Figure 7a. Since the temperature was increased, some of the electrons were excited by the thermal stimulus and left the external electric field. Thermal excitation causes electrons to move irregularly, which increases the impedance of the inductor and reduces the current generated by the electric field. Therefore, the amplitude of S11 gradually attenuates with the increasing temperature. Although the signal intensity at 900 °C is much weaker than at room temperature, the resonant frequency of the sensor can still be extracted. The result shown in Figure 7b indicates that the initial frequency of the sensor shifted uniformly toward a low frequency, and the frequency drift was 3.015 kHz/°C. Figure 7c shows the resonant frequency responses to the pressure (from approximately 0 kPa to 700 kPa) at 900 °C. The resonant frequency decreased from 113.201 MHz to 101.491 MHz, and the pressure sensitivity of the sensor changed to 16.72 kHz/kPa. Figure 7d shows the maximum error of frequency at different pressures in a 900 °C environment and demonstrates that the sensor is reasonable and reliable.

**Figure 8:**
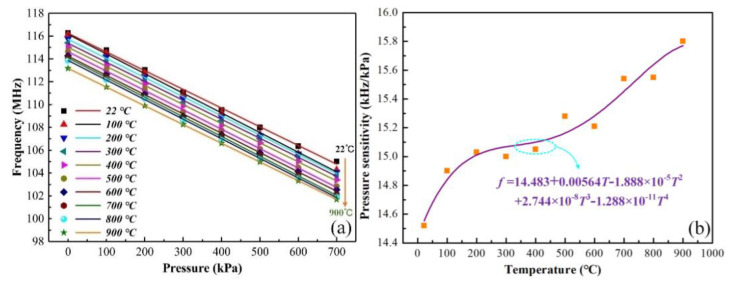
(**a**) The pressure versus resonant frequency curve at temperatures of 22–900 °C. (**b**) The pressure sensitivity of the sensor at different temperatures.

Further, the resonant frequency varied with the pressure of the sensor at different temperatures (from 22 °C to 900 °C, increment of 100 °C), as shown in Figure 8a. It can be seen that the resonant frequency changed linearly with the pressure within the range of 20 kPa to 700 kPa. The pressure sensitivity of the sensor was between 22 °C and 900 °C and is displayed in Figure 8b. It can be seen that the pressure sensitivity increased with the temperature. This was mainly due to the high temperature affecting the elastic modulus, the thermal expansion coefficient, and the dielectric constant of MgO. In addition, a wireless temperature sensor can be integrated with the pressure sensor for calibrating the pressure frequency drift.

## 5. Conclusions

In this work, an LC wireless passive pressure sensor based on a single-crystalline magnesium oxide (MgO) MEMS processing technique was proposed and experimentally demonstrated for applications in high-temperature environments up to 900 °C. The capacitor cavity was fabricated using a MgO MEMS technique. This MEMS processing technique, including the wet chemical etching and direct bonding process, can improve the operating temperature of the sensor. The experimental results show that the resonant frequency changed linearly with the pressure. With the temperature change from 22 °C to 900 °C, the pressure sensitivity of the sensor increased from 14.52 kHz/kPa to 16.72 kHz/kPa. This paper provides a batch-production and integrated manufacturing method of the pressure sensor for high-temperature applications, which helps to ensure the consistency of sensors, improve the production efficiency, and reduce costs. We think using single-crystal materials is one of the development trends for high-temperature sensors in the future.

## Figures and Tables

**Table 1:** Geometrical parameters of the designed LC wireless passive sensor.

Parameter	Symbol	Value (mm)
Thickness of the sensitive diaphragm	d_m_	0.124
Thickness of the support layer	d_t_	0.4
Length of the cavity	d_p_	0.076
Length of the sensor substrate	L	20
Radius of the cavity	r	4
Diameter of the capacitor	a	8
Width of inductance coils	w_c_	0.5
Space of inductance coils	w_g_	0.5
Thickness of the coils	d_s_	0.02

## Data Availability

Not applicable.
